# Percutaneous Coronary Intervention Versus Medical Therapy for Chronic Total Occlusion of Coronary Arteries: A Systematic Review and Meta-Analysis

**DOI:** 10.1007/s11883-019-0804-8

**Published:** 2019-08-09

**Authors:** Ka Hou Christien Li, Ka Hei Gabriel Wong, Mengqi Gong, Tong Liu, Guangping Li, Yunlong Xia, Jeffery Ho, Luis Nombela-Franco, Abhishek C. Sawant, Simon Eccleshall, Gary Tse, Vassilios S. Vassiliou

**Affiliations:** 1 Department of Medicine and Therapeutics, Faculty of Medicine, Chinese University of Hong Kong, Hong Kong, SAR People’s Republic of China; 2 Li Ka Shing Institute of Health Sciences, Faculty of Medicine, Chinese University of Hong Kong, Hong Kong, SAR People’s Republic of China; 3 Faculty of Medicine, Newcastle University, Newcastle, UK; 4 Tianjin Key Laboratory of Ionic-Molecular Function of Cardiovascular Disease, Department of Cardiology, Tianjin Institute of Cardiology, Second Hospital of Tianjin Medical University, Tianjin, 300211 People’s Republic of China; 5 Department of Cardiology, First Affiliated Hospital of Dalian Medical University, Dalian, China; 6 Department of Microbiology, The Chinese University of Hong Kong, Hong Kong, SAR People’s Republic of China; 7 Cardiology Department, Instituto Cardiovascular, Hospital Clínico San Carlos, IdISSC, Madrid, Spain; 8 Division of Interventional Cardiology, Banner University Medical Center, Phoenix, AZ USA; 9 Norfolk and Norwich University Hospital and Norwich Medical School, University of East Anglia, Norwich, UK; 10 Royal Brompton Hospital and Imperial College London, London, UK; 11 Bob Champion Research and Education, Second Floor, University of East Anglia, Norwich Research Park, Norwich, NR4 7TJ UK

**Keywords:** Chronic total occlusion, Mortality, Adverse outcomes

## Abstract

**Purpose of Review:**

Chronic total occlusion (CTO) of the coronary arteries is a significant clinical problem and has traditionally been treated by medical therapy or coronary artery bypass grafting. Recent studies have examined percutaneous coronary intervention (PCI) as an alternative option.

**Recent Findings:**

This systematic review and meta-analysis compared medical therapy to PCI for treating CTOs.

**Summary:**

PubMed and Embase were searched from their inception to March 2019 for studies that compared medical therapy and PCI for clinical outcomes in patients with CTOs. Quality of the included studies was assessed by Newcastle–Ottawa scale. The results were pooled by DerSimonian and Laird random- or fixed-effect models as appropriate. Heterogeneity between studies and publication bias was evaluated by *I*^2^ index and Egger’s regression, respectively. Of the 703 entries screened, 17 studies were included in the final analysis. This comprised 11,493 participants. Compared to PCI, medical therapy including randomized and observational studies was significantly associated with higher risk of all-cause mortality (risk ratio (RR) 1.99, 95% CI 1.38–2.86), cardiac mortality (RR 2.36 (1.97–2.84)), and major adverse cardiac event (RR 1.25 (1.03–1.51)). However, no difference in the rate of myocardial infarction and repeat revascularization procedures was observed between the two groups. Univariate meta-regression demonstrated multiple covariates as independent moderating factors for myocardial infarction and repeat revascularization but not cardiac death and all-cause mortality. However, when only randomized studies were included, there was no difference in overall mortality or cardiac death. In CTO, when considering randomized and observational studies, medical therapy might be associated with a higher risk of mortality and myocardial infarction compared to PCI treatment.

**Electronic supplementary material:**

The online version of this article (10.1007/s11883-019-0804-8) contains supplementary material, which is available to authorized users.

## Introduction

A chronic total occlusion (CTO) is characterized by the complete or near complete occlusion of a coronary artery with no or minimal downstream flow (TIMI flow grade 0 or 1) for a period longer than 3 months [1]. Among patients with coronary heart disease who are referred for coronary angiography, a large proportion ranging from 18 to 52% are found to have CTOs [2–4]. However, only a small proportion of these patients subsequently undergo percutaneous coronary intervention (PCI) [2, 5] with approximately one-tenth of CTO patients in North America undergoing PCI in the end [3, 6, 7]. Therefore, despite the rising popularity of percutaneous intervention for this group of CTO patients, the vast majority are either treated with medical therapy or coronary artery bypass grafting (CABG) [6]. However, it is recognized that patients with CTOs have a poorer prognosis when compared to those with coronary disease but without CTOs [8, 9]. This is often exacerbated by the fact that many patients with CTO tend to be asymptomatic, which leads to a delay in the diagnosis, investigations, and subsequent treatment [3] which may partly at least explain the broad prevalence range.

Hence, despite guidelines [1, 10, 11] recommending consideration of PCI in patients with CTO to improve survivability and quality of life, the prevalence of PCI in CTO patients remains low. This trend is potentially further reinforced by the 2016 EXPLORE trial [12]. Observation and randomized studies appear to disagree with the potential benefit of PCI [13–15, 16•, 17••, 18, 19••]. The issue thus of whether PCI or medical therapy should be the preferred management option in patients with CTOs remains controversial. This is also further compounded by potential sex-based differences in CTO management [20]. As such, this systematic review and meta-analysis seeks to combine all available cohort studies involving head-to-head comparison between PCI and medical therapy in CTO patients to offer a more comprehensive understanding. Further multivariate meta-regression analysis has also been used to help identify covariates that can potentially moderate outcome measures between the two interventions.

## Methods

### Search Strategy, Inclusion and Exclusion Criteria

This study was conducted according to the Preferred Reporting Items for Systematic Reviews and Meta-Analyses (PRISMA) statement. PubMed and Embase were searched for studies that compare medical therapy to PCI in patients with CTO. The following search terms were used for both databases: [(chronic total occlusion) AND (((percutaneous coronary intervention) OR (revascularization)) AND ((optimal medical therapy) OR (medical therapy)))]. The search period was from the beginning of the database through to March 1, 2019 without language restrictions. Both fully published studies and abstracts were used. The following inclusion criteria were used: (1) studies involving patients with CTO requiring either medical therapy or PCI; (2) measured and compared the difference in outcome between the two procedures, medical therapy and PCI. These outcomes assessed included all-cause mortality, cardiac death, cerebral vascular accident (CVA), myocardial infarction (MI), repeated revascularization, major adverse cardiac event (MACE), and major adverse cardiac and cerebrovascular events (MACCEs). MACE was defined as a composite of non-fatal stroke, non-fatal MI, and cardiovascular death, whereas MACCE was defined as a composite of all-cause mortality, MI, stroke, or ischemia-driven target vessel revascularization.

The Newcastle–Ottawa Quality Assessment Scale (NOS) and Cochrane risk of bias tool were used for quality assessment of the included studies. The NOS system evaluated the categories of study participant selection, results comparability, and quality of the outcomes. Specifically, the following characteristics were assessed: (1) representativeness of the exposed cohort; (2) selection of the non-exposed cohort; (3) ascertainment of exposure; (4) demonstration that outcome of interest was not present at the start of study; (5) comparability of cohorts based on study design or analysis; (6) assessment of outcomes; (7) follow-up periods that were sufficiently long for outcomes to occur; (8) adequacy of follow-up of cohorts. This scale varied from zero to nine stars, which indicated that studies were graded as poor quality if the score was < 5, fair if the score was 5 to 7, and good if the score was > 8. Studies with a score equal to or higher than 6 were included. The details of the NOS quality assessment and Cochrane risk of bias assessment for randomized controlled trials are shown in Supplementary Table 1 and Supplementary Fig. 1.

### Data Extraction and Statistical Analysis

Data from different studies were entered in pre-specified spreadsheets in Microsoft Excel. All potentially relevant studies were retrieved as complete manuscripts, which were assessed fully to determine their compliance with the inclusion criteria. We extracted the following data from the included studies: (1) publication details: last name of first author, publication year, and locations; (2) study design; (3) outcome(s); (4) characteristics of the population including sample size, gender, age, and number of subjects. Two reviewers (K.H.G.W. and K.H.C.L) reviewed each included study independently. Disagreements were resolved by adjudication with input from a third reviewer (G.T.).

Heterogeneity across studies was determined using Cochran’s *Q* value and the *I*^2^ statistic from the standard *χ*^2^ test. Cochran’s *Q* value is the weighted sum of squared differences between individual study effects and the pooled effect across studies. The *I*^2^ statistic from the standard *χ*^2^ test describes the percentage of variability in the effect estimates resulting from heterogeneity. *I*^2^ > 50% was considered to reflect significant statistical heterogeneity. The random-effects model using the inverse variance heterogeneity method was used with *I*^2^ > 50%. To locate the origin of the heterogeneity, sensitivity analysis excluding one study at a time was also performed. Funnel plots showing standard errors or precision against the logarithms of the odds ratio were constructed. The Begg and Mazumdar rank correlation test and Egger’s test were used to assess for possible publication bias. Possible associations between population co-variables and study outcomes were explored using multivariate meta-regression. To account for missing data, we used mean imputation (< 10% missing) or random imputation (> 10% missing). All statistical analysis was conducted using Review Manager 5.3 for MacOS and Comprehensive Meta-Analysis (CMA) version 3.0 (Biostat, Inc., Englewood, NJ, USA). Statistical significance was set as *P* value of less than 0.05.

## Results

### Patient Baseline Characteristics

**Fig. 1:**
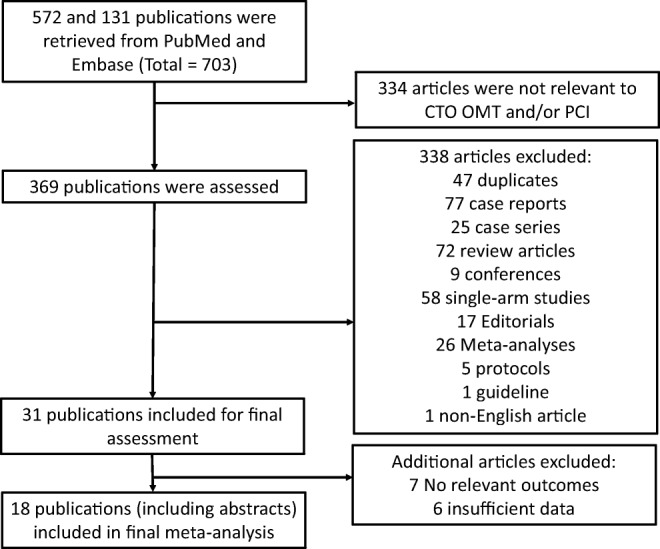
PRISMA flow diagram for the study selection process

A flow diagram detailing the search and study selection process is illustrated in Fig. 1. In the final meta-analysis, a total of 15 cohort studies and 3 randomized controlled trials involving 11,928 patients between 2011 and 2019 met our selection criteria for inclusion [13–15, 16•, 17••, 18, 19••, 21–31]. Two studies included the same population [22, 31] and therefore we only included the most recent one [31] reducing our number of studies to a total of 17, involving 11,493 patients. The mean age of the included population was 57.4 years, of the majority being male (81.8%). The baseline characteristics of all patients and follow-up duration based on individual studies are summarized in Table 1. However, only the baseline characteristics for 13 (out of 17) studies were available for inclusion in Table 1 [13, 14, 16•, 17••, 18, 19••, 21–25, 27, 29, 31]. This is due to the lack of sufficient data provided by the remaining four studies [15, 26, 28, 30]. Furthermore, the baseline characteristics included in three studies (*) utilized that of the overall population as the studies pooled the baseline characteristics of two or more intervention groups (e.g., CABG and PCI) together [23, 25, 27] (Table 1). Nonetheless, sufficient intervention-specific data was still provided by all 17 studies for effective pooling of outcome measures. Outcome measures pooled in this meta-analysis include (1) all-cause mortality, (2) cardiac death, (3) CVA/stroke, (4) MI, (5) repeat revascularization, (6) MACE, and (7) MACCE.

**Table 1:** Baseline characteristic of patients from the included studies

Study	Sample size (*n*)	Age(mean ± SD)	Male *n* (%)	HTN *n* (%)	DM *n* (%)	Dyslipidemia *n* (%)	MI Hx *n* (%)	Smoking *n* (%)	LAD lesions *n* (%)	Prior PCI *n* (%)	Calcification *n* (%)	Mean follow-up (months)
Kim 2015	224	64.4 ± 11.8	188 (83.9)	145 (64.7)	112 (50)	62 (27.7)	75 (33.5)	83 (37.1)	124 (55.4)	55 (24.6)	50 (22.3)	Median: 46.5
Jang 2015*	738	62.9 ± 10.8	609 (82.5)	475 (64.4)	346 (46.9)	233 (31.6)	– (–)	216 (29.3)	268 (36.3)	149 (20.2)	146 (25.7)	Median: 42
Shuvy 2017*	2279	65.7 ± 10.2	1839 (80.7)	1841 (80.8)	1060 (46.5)	1841 (80.8)	683 (30)	689 (30.2)	– (–)	– (–)	– (–)	26.6
Tomasello 2015	1602	68.6 ± 11.6	1348 (84.1)	1249 (78)	477 (29.8)	1009 (63)	690 (43.1)	708 (44.2)	– (–)	506 (31.6)	188 (11.7)	12
Ladwiniec 2015	1056	64.8 ± 10.5	807 (76.4)	574 (54.4)	215 (20)	– (–)	596 (56.4)	739 (70)	143 (13.5)	86 (8.1)	– (–)	60
Yang 2016	1363	63.6 ± 11.0	1222 (89.7)	986 (72.3)	702 (50)	447 (32.8)	391 (28.7)	467 (34.3)	541 (39.7)	386 (28.3)	256 (18.8)	Median: 45.9
Choy SY 2017	640	64.1 ± 11.0	472 (73.8)	423 (66.1)	285 (40)	198 (30.9)	128 (20)	356 (55.6)	451 (70.5)	– (–)	– (–)	60
Guo 2018	326	64.5 ± 10.2	241 (73.9)	226 (69.3)	106 (32.5)	172 (52.8)	94 (28.8)	149 (45.7)	115 (35.3)	29 (8.9)	104 (31.9)	47.2
Yuste 2017*	1248	67.3 ± 10.9	1048 (84)	911 (73)	524 (42)	824 (66)	400 (32.1)	674 (54)	– (–)	– (–)	– (–)	42
Choo 2019	898	63.9 ± 11.3	637 (70.9)	546 (60.8)	401 (44.7)	– (–)	– (–)	211 (23.5)	318 (35.4)	– (–)	– (–)	Median: 26.4
Lee SW 2019 (DECISION-CTO)	815	62.5 ± 10.1	657 (80.6)	496 (60.9)	265 (32.5)	463 (56.8)	79 (9.7)	227 (27.9)	344 (42.2)	136 (16.7)	– (–)	60
Werner 2018 (EUROCTO)	396	65 ± 9.8	333 (84.1)	287 (72.4)	125 (31.6)	321 (81.1)	84 (21.2)	282 (71.2)	104 (26.3)	216 (54.5)	147 (37.1)	12
Mashayekhi 2018 (REVASC)	205	Median: PCI (65), OMT (68)	181 (88.3)	174 (84.9)	63 (30.7)	– (–)	77 (37.6)	49 (23.9)	40 (19.5)	61 (29.8)	72 (35.1)	12

### Medical Therapy Versus PCI in CTO Patients: All-Cause Mortality

**Fig. 2:**
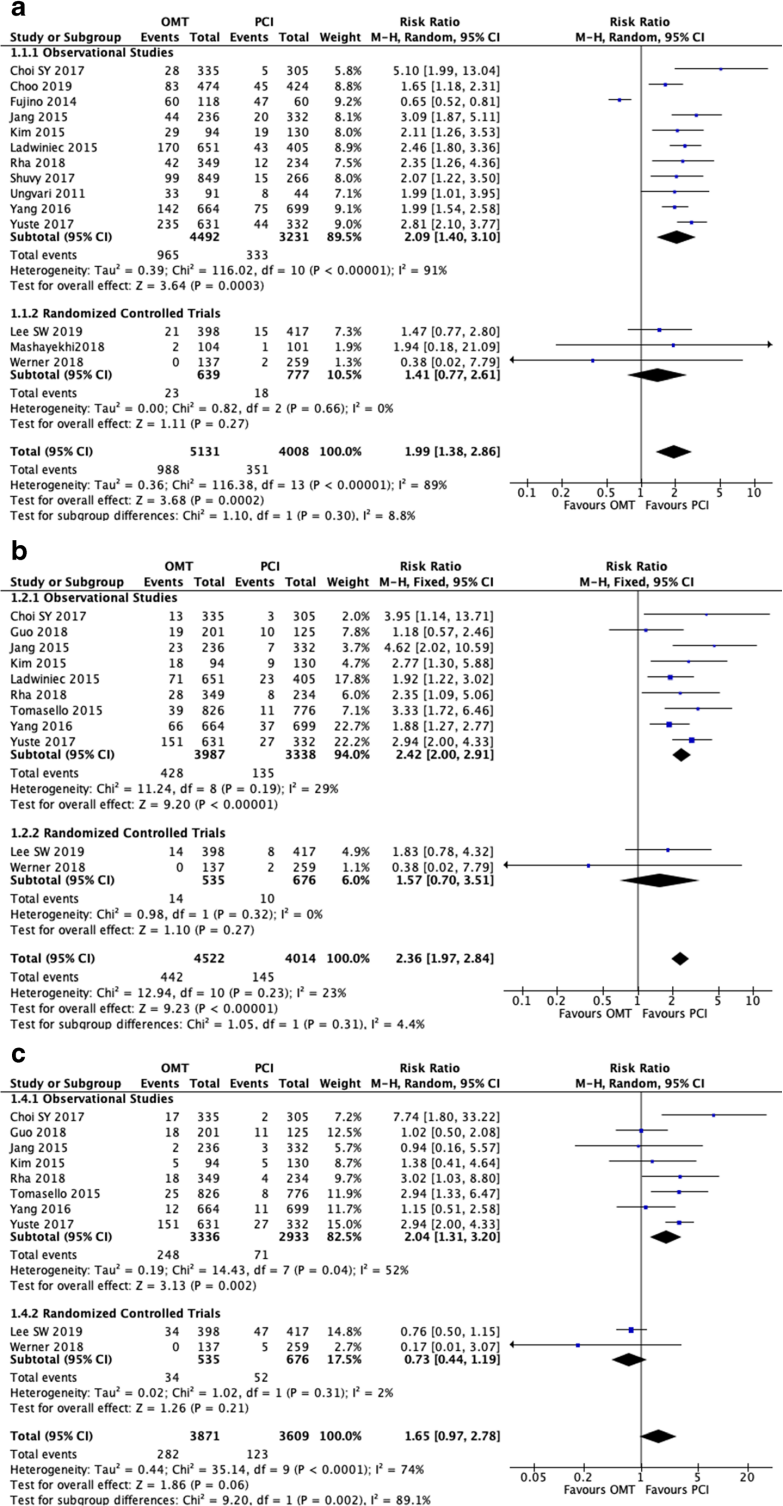

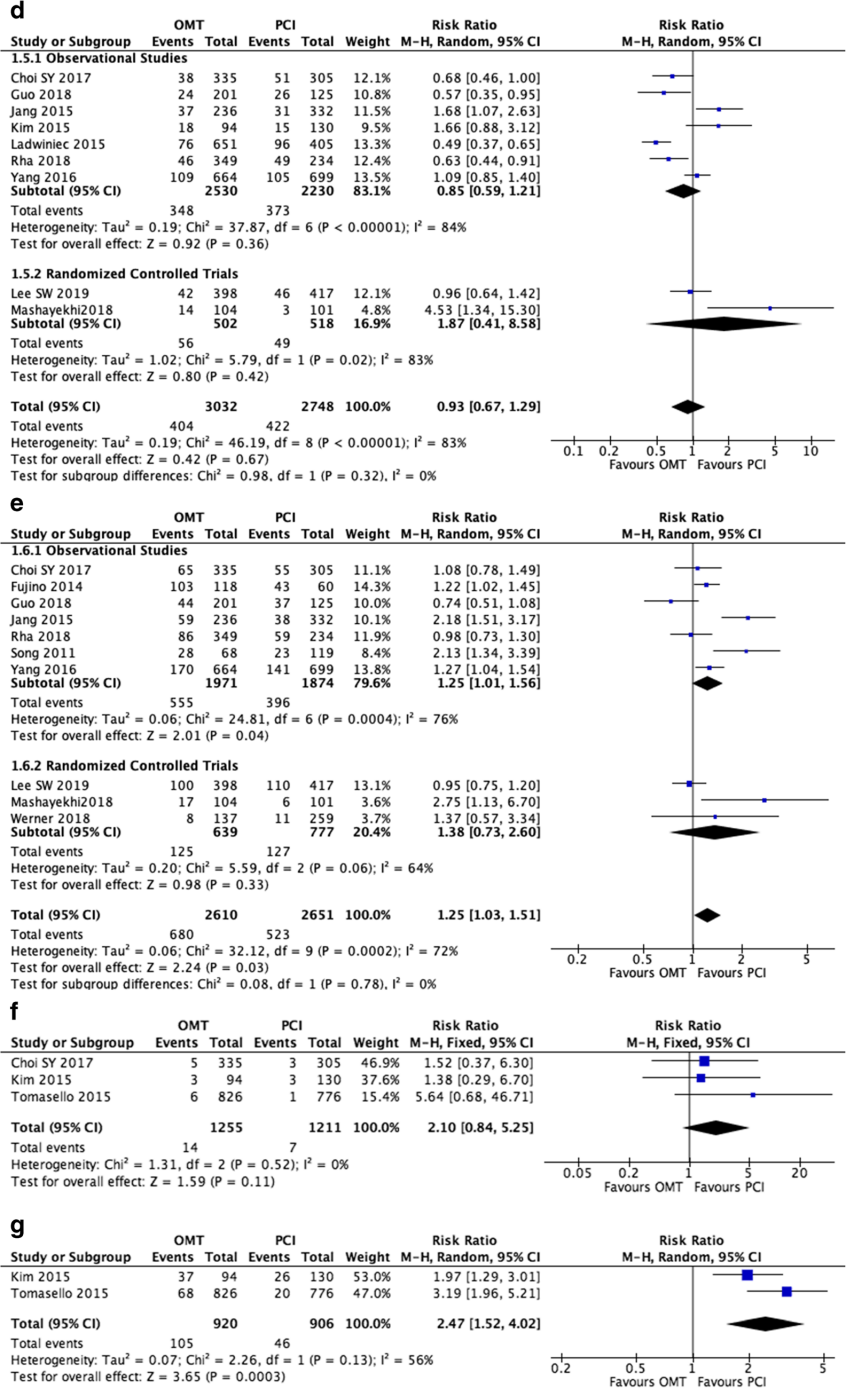
(**a**) Forest plots comparing risk of all-cause mortality events between OMT and PCI in patients with CTO. (**b**) Forest plots comparing risk of cardiac death between OMT and PCI in patients with CTO. (**c**) Forest plots comparing risk of CVA/stroke events between OMT and PCI in patients with CTO. (**d**) Forest plots comparing risk of myocardial infarction between OMT and PCI in patients with CTO. (**e**) Forest plots comparing risk of repeated revascularization events between OMT and PCI in patients with CTO. (**f**) Forest plots comparing risk of MACE events between OMT and PCI in patients with CTO. (**g**) Forest plots comparing risk of MACCE events between OMT and PCI in patients with CTO

**Fig. 3:**
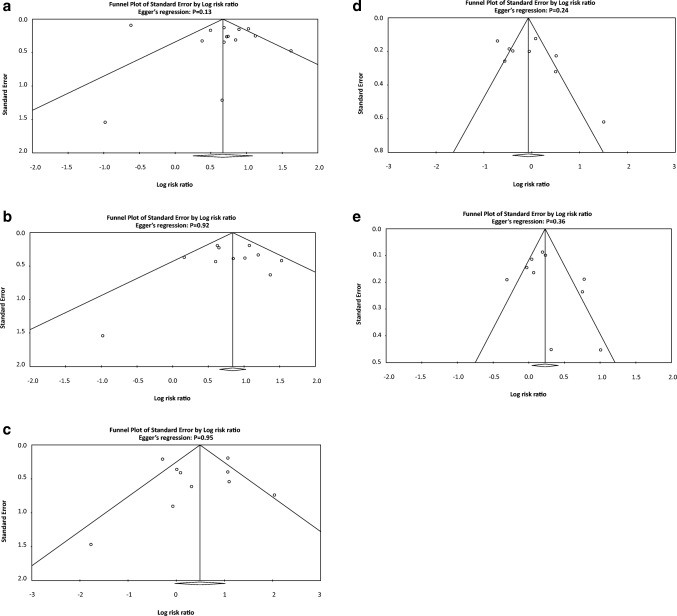
(**a**) Trim-and-fill funnel plots with Egger’s regression test of all-cause mortality comparing between OMT and PCI in patients with CTO. (**b**) Trim-and-fill funnel plots with Egger’s regression test of cardiac death comparing between OMT and PCI in patients with CTO. (**c**) Trim-and-fill funnel plots with Egger’s regression test of CVA/stroke comparing between OMT and PCI in patients with CTO. (**d**) Trim-and-fill funnel plots with Egger’s regression test of MI comparing between OMT and PCI in patients with CTO. (**e**) Trim-and-fill funnel plots with Egger’s regression test of repeated revascularization comparing between OMT and PCI in patients with CTO

A total of 14 out of 17 studies reported all-cause mortality in CTO patients after medical therapy or PCI [13–15, 16•, 17••, 18, 19••, 23–27, 30, 31]. Of these, only two studies reported in favor of medical therapy [15, 19••] while the remaining 12 reported in favor of PCI. Pooled analysis of all the included studies demonstrated that patients with CTO treated with medical therapy have a significantly higher risk of all-cause mortality when compared to the PCI group (RR 1.99, 95% CI 1.38–2.86, *P* = 0.0002; Fig. 2a). However, what is important to recognize here is that there is a significant disagreement with regard to the observational and randomized studies. The observational studies significantly favor PCI (RR 2.09, 1.40–3.10, *P* = 0.0003) while the randomized studies showed a non-significant improvement in mortality (RR 1.41, 0.77–2.61, *P* = 0.27), indicating that perhaps the current randomized studies do not have sufficient power to confirm a benefit, which is smaller than what is observed in the cohort studies. *I*^2^ was 89% across all studies, indicating a high degree of heterogeneity. In order to locate the origin of the heterogeneity, sensitivity analysis excluding one study at a time was performed. Doing so did not significantly alter the overall heterogeneity. In addition, the results of Egger’s test showed no evidence of publication bias (Egger’s regression test *P* = 0.13; Fig. 3a).

### Medical Therapy Versus PCI in CTO Patients: Cardiac Mortality

A total of 11 out of 17 studies reported cardiac mortality in CTO patients [13, 16•, 17••, 19••, 21, 23–26, 29, 31]. All studies included favored the use of PCI apart from Werner et al. [19••]. Pooled analysis of the included studies demonstrated that patients with CTO treated medical therapy had significantly higher risk of cardiac mortality when compared to the use of PCI (RR 2.36, 95% CI 1.97–2.84, *P* < 0.00001; Fig. 2b). However, the discrepancy between observation and randomized studies existed. While in observation studies there was a very positive improvement seen with PCI (RR 2.42, 95% CI 2.00–2.91, *P* < 0.00001), there was no significant difference observed with the randomized controlled trials (RCTs) (RR 1.57, 95% CI 0.70–3.51, *P* = 0.27). Nonetheless, the relative risk of 1.57 could still suggest that the RCT even when pooled remained underpowered. *I*^2^ was 23% across all studies, indicating a low degree of heterogeneity. Furthermore, results from Egger’s test showed no evidence of publication bias (Egger’s regression test *P* = 0.92; Fig. 3b).

### Medical Therapy Versus PCI in CTO Patients: Myocardial Infarction

A total of 10 studies reported MI as an outcome in CTO patients undergoing either medical therapy or PCI [13, 17••, 19••, 21, 23–26, 29, 31]. Three out of 10 studies reported in favor of medical therapy [17••, 19••, 23]. Pooled analysis of the included studies showed that medical therapy was not significantly associated with a higher risk of MI when compared to the PCI group (RR 1.65, 95% CI 0.97–2.78, *P* = 0.06; Fig. 2c). However, when only RCTs were considered, medical therapy showed a non-statistical improvement over PCI (RR 0.73, 95% CI 0.44–1.19, *P* = 0.21). Significance was only achieved during pooled analysis of observational studies, favoring the PCI group (RR 2.04, 95% CI 1.31–3.20, *P* = 0.002). *I*^2^ was 74% across all studies, indicating a high degree of heterogeneity. Sensitivity analysis excluding one study at a time was performed to locate the origin of the heterogeneity which did not significantly alter the overall heterogeneity. Results from the Egger’s test showed no evidence of publication bias (Egger’s regression test *P* = 0.95; Fig. 3c).

### Medical Therapy Versus PCI in CTO Patients: Repeated Revascularization

A total of nine studies were included for reporting repeated revascularization in CTO patients with either medical therapy or PCI [13, 16•, 17••, 18, 21, 23, 24, 26, 31]. Of these, five studies reported in favor of medical therapy [13, 16•, 17••, 21, 26]. A pooled analysis of the included studies demonstrated that the use of medical therapy is associated with lower risk of repeat revascularization (RR 0.93, 95% CI 0.67–1.29, *P* = 0.67; Fig. 2d). While observational studies showed a non-statistical preference for OMT (RR 0.85, 95% CI 0.59–1.21, *P* = 0.36), the randomized studies showed a non-statistical reduction of revascularization with PCI (RR 1.87, 95% CI 0.41–8.58, *P* = 0.42). *I*^2^ was 84% across all studies, indicating a high degree of heterogeneity. Sensitivity analysis excluding one study at a time was performed to locate the origin of the heterogeneity, which did not significantly alter the overall heterogeneity. Results from Egger’s test showed no evidence of publication bias (Egger’s regression test *P* = 0.24; Fig. 3d).

### Medical Therapy Versus PCI in CTO Patients: Major Adverse Cardiovascular Event

A total of 10 studies reported MACE in CTO patients [13, 15, 17••, 18••, 19••, 21, 23, 26, 28, 31]. Of these, only three out of eight studies supported the use of medical therapy. A pooled analysis of the included studies illustrated that the use of PCI tended to lower risk of MACE when compared to medical therapy (RR 1.25, 95% CI 1.03–1.51, *P* = 0.03; Fig. 2e). Both observational studies (RR 1.25, 95% CI 1.01–1.56, *P*= 0.0004 ) and randomized trials (RR 1.38, 95% 0.73–2.60, *P* = 0.33) favored PCI in terms of MACE outcomes. Furthermore, *I*^2^ was 76% across all studies, indicating a high degree of heterogeneity. Sensitivity analysis excluding one study at a time was performed to locate the origin of the heterogeneity. Doing so did not significantly alter the overall heterogeneity. However, results from Egger’s test showed no evidence of publication bias (Egger’s regression test *P* = 0.36; Fig. 3e).

### Medical Therapy Versus PCI in CTO Patients: Cerebral Vascular Accident/Stroke

Regarding CVA/stroke, only 3 out of 14 studies were found to report this outcome in CTO patients [13, 24, 29]. All three studies reported in favor of PCI over medical therapy. A pooled analysis further supported this, showing that medical therapy has more than twice the risk of causing a CVA/stroke compared to PCI (RR 2.10, 95% CI 0.84–5.25, *P* = 0.11; Fig. 2f). However, this result was not statistically significant. *I*^2^ was 0% across all studies, indicating a lack of heterogeneity. Results from Egger’s test showed no evidence of publication bias (Egger’s regression test *P* = 0.22).

### Medical Therapy Versus PCI in CTO Patients: MACCE

Lastly, the outcome of MACCE was only reported in two studies [24, 29]. Both studies individually favored the use of PCI to avoid such events, which was supported by a pooled analysis (RR 2.47, 95% CI 1.52–4.02, *P* = 0.0003; Fig. 2g). *I*^2^ was 56% across all studies, indicating a moderate level of heterogeneity. However, an exclude-one sensitivity analysis could not be done, as there are only two studies involved. Similarly, Begg’s and Egger’s analysis could not be used due to the limited number of studies involved.

### Univariate Meta-Regression Analysis of All Outcome Measures

A univariate meta-regression analysis was conducted using all common covariates across the 17 studies included for four outcome measures, which were all-cause mortality, cardiac death, MI, and repeat revascularization. CVA/stroke, MACE, and MACCE outcomes were omitted from regression analysis due to a lack of reporting from relevant articles. Covariates used were mean age, male sex, hypertension, diabetes mellitus, dyslipidemia, smoking, prior PCI, LAD lesion, and calcification. Results of the meta-regression are shown in Table 2 accordingly. No variables were found to independently moderate all-cause mortality and cardiac death outcomes. However, mean age, hypertension, smoking, LAD lesion, and the presence of calcification were also found to be a significant moderator of MI while repeat revascularization was moderated by male gender, diabetes mellitus, smoking, and prior PCI. Apart from these, none of the other factors moderated cardiac death, MI, repeat revascularization, or all-cause mortality outcomes. Slope coefficients did not differ significantly from zero (*P* > 0.05).

**Table 2:** Multivariate meta-regression analysis of outcome measures

Univariate meta-regression						
Variable	Slope coefficient	SE	*Z* value	*P* value	95% CI	
					Lower limit	Upper limit
All-cause mortality						
Mean age (years)	− 0.0601	0.109	− 0.553	0.580	− 0.273	0.153
Male gender	− 0.969	3.102	− 0.312	0.755	− 7.048	5.111
Hypertension	− 1.628	1.849	− 0.881	0.378	− 5.251	1.995
Diabetes mellitus	0.0376	1.558	0.0241	0.981	− 3.016	3.091
Dyslipidemia	− 1.086	0.877	− 1.239	0.215	− 2.804	0.632
Smoking	0.933	1.082	0.863	0.388	− 1.187	3.054
Cardiac death						
Mean age (years)	0.0799	0.0523	1.529	0.126	− 0.0225	0.182
Male gender	1.049	1.877	− 2.629	0.576	− 2.629	4.728
Hypertension	1.057	1.262	0.837	0.402	− 1.417	3.532
Diabetes mellitus	0.692	0.893	0.775	0.438	− 1.058	2.443
Dyslipidemia	0.0645	0.613	0.105	0.916	− 1.137	1.266
Myocardial infarction	− 0.0875	0.810	− 0.108	0.914	− 1.674	1.499
Myocardial infarction						
Mean age (years)*	0.257	0.0517	4.969	< 0.0001	0.155	0.358
Male gender	− 1.504	5.000	− 0.301	0.764	− 11.301	8.293
Hypertension*	8.026	2.275	3.528	0.000419	3.567	12.486
Diabetes mellitus	1.106	3.115	0.355	0.722	− 4.999	7.212
Smoking*	4.611	1.023	4.506	< 0.0001	2.605	6.617
LAD lesion*	4.907	2.481	1.978	0.0480	0.0440	9.769
Calcification*	− 6.476	3.751	− 1.726	0.0843	− 13.828	0.876
Repeat revascularization						
Mean age (years)	− 0.01	0.133	− 0.751	0.452	− 0.361	0.161
Male gender*	6.288	2.243	2.803	0.00506	1.891	10.684
Hypertension	− 2.913	2.107	1.383	0.167	− 1.216	7.043
Diabetes mellitus*	2.808	1.0142	2.769	0.00563	0.820	4.796
Smoking*	− 2.388	0.605	− 3.950	< 0.0001	− 3.573	− 1.203
Prior PCI*	4.151	1.775	2.339	0.0193	0.673	7.630

## Discussion

This systematic review and meta-analysis included 17 cohort studies with a total of 11,493 patients, comparing the use of medical therapy and PCI in patients with known CTO. A total of seven outcomes including all-cause mortality, cardiac death, MI, repeat revascularization, MACE, CVA/stroke, and MACCE were assessed. A subsequent univariate meta-regression was also conducted to evaluate the impact of covariates on outcome measures. Our pooled analysis found a statistically significant association between PCI and lower risk of all-cause mortality, cardiac death, MI, MACE, and MACCE when compared to medical therapy. However, this was driven predominantly by the observational cohorts. The randomized studies showed a potential improvement with PCI in overall mortality, cardiac death, repeat revascularization, and MACE; however, no statistical significance was achieved. PCI tended to reduce CVA/stroke outcomes when compared to medical therapy, but the results did not reach significance, which may be due to the limited sample size. On the other hand, MACE which included admission for heart failure and non-fatal strokes was favored by PCI. A non-significant reduced risk of repeat revascularization was found to be associated with medical therapy (*P* > 0.05). This is likely due to further activation of the inflammatory pathway, which leads to the development of intimal hyperplasia and subsequent restenosis following PCI [32]. In our univariate regression analysis, there were no significant moderators of all-cause mortality and cardiac death. This is not in keeping with a large Swedish study involving 14,441 patients, which reported that presence of a CTO was associated with the highest risk of mortality in patients less than 60 years of age compared to the low risk found in octogenarians [33]. However, the lack of association between diabetes and sex is supported by another study [33]. Interestingly, our univariate analysis also showed hypertension, dyslipidemia, and smoking to be non-significantly linked with all-cause mortality. As for MI, it is surprising that diabetes mellitus was found to be a non-significant moderator given the extensive literature supporting this correlation [34] while mean age, hypertension, smoking, having LAD lesions, and calcification were significant moderators.

Our findings differ from those of a previous meta-analysis comparing medical therapy and PCI in patients with stable coronary artery disease. The latter reported that PCI was not significantly better than optimal medical therapy in reducing risk of all-cause mortality, cardiac death, and MI [35]. Similar findings were noted in the landmark Courage Trial where PCI was not found to be superior to medical therapy but CTO patients were not represented in this study and patients would receive more intense medical therapy than what would be expected in reality [36]. Another meta-analysis confirmed that successful PCIs in CTO patients pertain to higher long-term survival along with reduced risk of developing subsequent MI [37]. Recently, a similar meta-analysis looking only into five articles stated that the PCI was significantly associated with reduced risk of all-cause mortality, cardiac death, and MACE in CTO patients. This result was further supported in their “infarct-related area” subgroup analysis [38]. In contrast, our meta-analysis utilized 17 studies with a significantly greater patient population. As such, our results favoring PCI in CTO should be interpreted in the knowledge that this is driven mainly by the outcomes in the observational cohorts and that the RCT failed to reach significance. Nonetheless, even in the RCT, a non-significant trend in benefit was seen with PCI groups indicating perhaps that more RCTs are needed for a definite answer. Despite this, our meta-analysis suggests that at the very least, there is no evidence to advice against PCI in CTO at present and possibly there might be some benefit.

## Limitations

A moderate to high degree of heterogeneity was identified during analysis of some outcome measures and we thus performed an exclude-one sensitivity analysis to locate the source of heterogeneity and rectify this. In addition, one of the areas of growing interest in CTO PCI work is to appropriately select patients by demonstrating objective evidence of reversible ischemia corresponding to the territory of the CTO, rather than simply relying on angina as a marker for this [39]. Unfortunately, the inclusion criteria from the studies used included “angina or reversible ischemia.” Therefore, we were not able to consider a specific subgroup of patients who had evidence of reversible ischemia on functional testing and viable myocardium. Finally, we have considered the observational and randomized studies together in the analysis but also separately. Although the observational studies favored the PCI quite significantly, this was not statistically seen when only the fewer randomized studies were considered; this indicates, however, that a true beneficial effect could well have been observed if more randomized studies were available to meta-analyze.

## Future Directions

The unmet clinical question is whether randomized studies in patients who have objective evidence of reversible ischemia, which can also be quantified, might benefit from PCI. Although it can be expected that patients with a higher burden of reversible ischemia and higher percentage of viable myocardium are likely to benefit more from percutaneous intervention, new evidence is needed to answer this question [40].

## Conclusions

The use of PCI in patients with CTO was found to be associated with lower risk of all outcome measures except repeat revascularization, a result driven predominantly by the observational cohorts as the randomized studies appeared to show a smaller benefit but were underpowered to statistically confirm that. However, statistically significant results were seen only for all-cause mortality, cardiac death, MI, MACE, and MACCE in pooled analysis and not in the RCT-specific subgroup analysis. Our results suggest that PCI could therefore be considered in the CTO in patients with angina or evidence of reversible ischemia. Larger randomized clinical trials can address an individualized personalized approach incorporating the risk and complexity associated with CTO PCI, the burden of ischemia, and myocardial viability.

## Electronic supplementary material

Supplementary Figure 1 Cochrane risk of bias assessment for included randomized controlled trials. (PNG 2742 kb)

High resolution image (TIFF 4918 kb)

Supplementary Table 1 (DOCX 24 kb)

## References

[1] Windecker S, Kolh P, Alfonso F, Collet JP, Cremer J, Falk V (2014). 2014 ESC/EACTS guidelines on myocardial revascularization: the task force on myocardial revascularization of the European Society of Cardiology (ESC) and the European Association for Cardio-Thoracic Surgery (EACTS) developed with the special contribution of the European Association of Percutaneous Cardiovascular Interventions (EAPCI). Eur Heart J.

[2] Christofferson RD, Lehmann KG, Martin GV, Every N, Caldwell JH, Kapadia SR (2005). Effect of chronic total coronary occlusion on treatment strategy. Am J Cardiol.

[3] Fefer P, Knudtson ML, Cheema AN, Galbraith PD, Osherov AB, Yalonetsky S, Gannot S, Samuel M, Weisbrod M, Bierstone D, Sparkes JD, Wright GA, Strauss BH (2012). Current perspectives on coronary chronic total occlusions: the Canadian Multicenter Chronic Total Occlusions Registry. J Am Coll Cardiol.

[4] Jeroudi OM, Alomar ME, Michael TT, El Sabbagh A, Patel VG, Mogabgab O (2014). Prevalence and management of coronary chronic total occlusions in a tertiary Veterans Affairs hospital. Catheter Cardiovasc Interv.

[5] Brilakis ES, Banerjee S, Karmpaliotis D, Lombardi WL, Tsai TT, Shunk KA, Kennedy KF, Spertus JA, Holmes DR, Grantham JA (2015). Procedural outcomes of chronic total occlusion percutaneous coronary intervention: a report from the NCDR (National Cardiovascular Data Registry). JACC Cardiovasc Interv.

[6] Azzalini L, Jolicoeur EM, Pighi M, Millan X, Picard F, Tadros VX (2016). Epidemiology, management strategies, and outcomes of patients with chronic total coronary occlusion. Am J Cardiol.

[7] Anderson HV, Shaw RE, Brindis RG, Hewitt K, Krone RJ, Block PC, McKay CR, Weintraub WS (2002). A contemporary overview of percutaneous coronary interventions. The American College of Cardiology-National Cardiovascular Data Registry (ACC-NCDR). J Am Coll Cardiol.

[8] Werner GS, Gitt AK, Zeymer U, Juenger C, Towae F, Wienbergen H, Senges J (2009). Chronic total coronary occlusions in patients with stable angina pectoris: impact on therapy and outcome in present day clinical practice. Clin Res Cardiol.

[9] Chi WK, Gong M, Bazoukis G, Yan BP, Letsas KP, Liu T, et al. Impact of coronary artery chronic total occlusion on arrhythmic and mortality outcomes. A systematic review and meta-analysis. JACC Clin Electrophysiol. 2018;4(9):1214-1223 10.1016/j.jacep.2018.06.011 30236396

[10] Levine GN, Bates ER, Blankenship JC, Bailey SR, Bittl JA, Cercek B, Chambers CE, Ellis SG, Guyton RA, Hollenberg SM, Khot UN, Lange RA, Mauri L, Mehran R, Moussa ID, Mukherjee D, Nallamothu BK, Ting HH (2011). 2011 ACCF/AHA/SCAI guideline for percutaneous coronary intervention: a report of the American College of Cardiology Foundation/American Heart Association Task Force on Practice Guidelines and the Society for Cardiovascular Angiography and Interventions. Circulation..

[11] Kearney K, Hira RS, Riley RF, Kalyanasundaram A, Lombardi WL (2017). Update on the management of chronic total occlusions in coronary artery disease. Curr Atheroscler Rep.

[12] Henriques JP, Hoebers LP, Ramunddal T, Laanmets P, Eriksen E, Bax M (2016). Percutaneous intervention for concurrent chronic total occlusions in patients with STEMI: the EXPLORE trial. J Am Coll Cardiol.

[13] Choi SY, Choi BG, Rha SW, Baek MJ, Ryu YG, Park Y, et al. Percutaneous coronary intervention versus optimal medical therapy for chronic total coronary occlusion with well-developed collaterals. J Am Heart Assoc. 2017;13;6(9). 10.1161/JAHA.117.006357 PMC5634287 28903939

[14] Choo EH, Koh YS, Seo SM, Lee JM, Kim HY, Park HJ, Kim PJ, Chang K, Jeon DS, Kim DB, Her SH, Park CS, Yoo KD, Chung WS, Seung KB (2019). Comparison of successful percutaneous coronary intervention versus optimal medical therapy in patients with coronary chronic total occlusion. J Cardiol.

[15] Fujino M, Ishihara M, Honda S, Kawakami S, Yamane T, Nagai T, et al. Long-Term Follow-Up of Patients with Acute Myocardial Infarction with Chronic Total Occlusion in the Non-Infarct Related Artery. Circulation. 2014;130(suppl_2).

[16] Ladwiniec A, Allgar V, Thackray S, Alamgir F, Hoye A (2015). Medical therapy, percutaneous coronary intervention and prognosis in patients with chronic total occlusions. Heart.

[17] Lee Seung-Whan, Lee Pil Hyung, Ahn Jung-Min, Park Duk-Woo, Yun Sung-Cheol, Han Seungbong, Kang Heejun, Kang Soo-Jin, Kim Young-Hak, Lee Cheol Whan, Park Seong-Wook, Hur Seung Ho, Rha Seung-Woon, Her Sung-Ho, Choi Si Wan, Lee Bong-Ki, Lee Nae-Hee, Lee Jong-Young, Cheong Sang-Sig, Kim Moo Hyun, Ahn Young-Keun, Lim Sang Wook, Lee Sang-Gon, Hiremath Shirish, Santoso Teguh, Udayachalerm Wasan, Cheng Jun Jack, Cohen David J., Muramatsu Toshiya, Tsuchikane Etsuo, Asakura Yasushi, Park Seung-Jung (2019). Randomized Trial Evaluating Percutaneous Coronary Intervention for the Treatment of Chronic Total Occlusion. Circulation.

[18] Mashayekhi K, Nuhrenberg TG, Toma A, Gick M, Ferenc M, Hochholzer W (2018). A randomized trial to assess regional left ventricular function after stent implantation in chronic total occlusion: the REVASC trial. JACC Cardiovasc Interv.

[19] Werner GS, Martin-Yuste V, Hildick-Smith D, Boudou N, Sianos G, Gelev V (2018). A randomized multicentre trial to compare revascularization with optimal medical therapy for the treatment of chronic total coronary occlusions. Eur Heart J.

[20] Cheney A, Kearney KE, Lombardi W (2018). Sex-based differences in chronic total occlusion management. Curr Atheroscler Rep.

[21] Guo Lei, Zhong Lei, Chen Kun, Wu Jian, Huang Rong-Chong (2018). Long-term clinical outcomes of optimal medical therapy vs. successful percutaneous coronary intervention for patients with coronary chronic total occlusions. Hellenic Journal of Cardiology.

[22] Hwang JW, Yang JH, Choi SH, Hwang JK, Jang WJ, Hahn JY, Song YB, Choi JH, Lee SH, Gwon HC (2016). Optimal medical therapy may be a better initial strategy in patients with chronic total occlusion of a single coronary artery. Int J Cardiol.

[23] Jang WJ, Yang JH, Choi SH, Song YB, Hahn JY, Choi JH, Kim WS, Lee YT, Gwon HC (2015). Long-term survival benefit of revascularization compared with medical therapy in patients with coronary chronic total occlusion and well-developed collateral circulation. JACC Cardiovasc Interv.

[24] Kim BS, Yang JH, Jang WJ, Song YB, Hahn JY, Choi JH, Kim WS, Lee YT, Gwon HC, Lee SH, Choi SH (2015). Clinical outcomes of multiple chronic total occlusions in coronary arteries according to three therapeutic strategies: bypass surgery, percutaneous intervention and medication. Int J Cardiol.

[25] Martin Yuste V, Gonzalez IF, Flores E, Hernandez M, Vazquez S, Robles C, Pernigotti A, Freixa X, Regueiro A, Brugaletta S, Sabate M (2017). TCT-564 Monocenter registry of 1248 consecutive patients with a coronary chronic total occlusion: predictor factors of cardiac death. J Am Coll Cardiol.

[26] Rha S-W, Choi SY, Choi BG, Byun JK, Mashaly A, Park Y, Jang WY, Kim WH, Park EJ, Choi JY, Na JO, Choi CU, Lim HE, Kim EJ, Park CG, Seo HS, Oh DJ (2018). Impact of successful percutaneous coronary intervention on chronic total occlusion outcomes in multi-vessel disease patients. J Am Coll Cardiol.

[27] Shuvy M, Qiu F, Chee ATA, Graham JJ, Abuzeid W, Buller C (2017). Management of chronic total coronary occlusion in stable ischemic heart disease by percutaneous coronary intervention versus coronary artery bypass grafting versus medical therapy. Am J Cardiol.

[28] Song M, Yun HE, Lee SH, Lee SR, Rhee KS, Kim WH (2011). Syntax score can predict long-term clinical outcomes in patients with chronic total occlusion. J Am Coll Cardiol.

[29] Tomasello SD, Boukhris M, Giubilato S, Marza F, Garbo R, Contegiacomo G (2015). Management strategies in patients affected by chronic total occlusions: results from the Italian registry of chronic total occlusions. Eur Heart J.

[30] Ungvari T, Ismail H, Shah N, Khan F, Ahmed D, Loh H (2011). Impact of therapy (medical versus revascularization) on the long-term survival of patients with coronary disease including a chronic total occlusion. Am J Cardiol.

[31] Yang JH, Kim BS, Jang WJ, Ahn J, Park TK, Song YB, Hahn JY, Choi JH, Lee SH, Gwon HC, Choi SH (2016). Optimal medical therapy vs. percutaneous coronary intervention for patients with coronary chronic total occlusion—a propensity-matched analysis. Circulation.

[32] Toutouzas K, Colombo A, Stefanadis C (2004). Inflammation and restenosis after percutaneous coronary interventions. Eur Heart J.

[33] Råmunddal T, Hoebers LP, Henriques JPS, Dworeck C, Angerås O, Odenstedt J (2016). Prognostic impact of chronic total occlusions: a report from SCAAR (Swedish Coronary Angiography and Angioplasty Registry). JACC Cardiovasc Interv.

[34] Chiha M, Njeim M, Chedrawy EG (2012). Diabetes and coronary heart disease: a risk factor for the global epidemic. Int J Hypertens.

[35] Pursnani S, Korley F, Gopaul R, Kanade P, Chandra N, Shaw RE, Bangalore S (2012). Percutaneous coronary intervention versus optimal medical therapy in stable coronary artery disease: a systematic review and meta-analysis of randomized clinical trials. Circ Cardiovasc Interv.

[36] Boden WE, O'Rourke RA, Teo KK, Hartigan PM, Maron DJ, Kostuk WJ, Knudtson M, Dada M, Casperson P, Harris CL, Chaitman BR, Shaw L, Gosselin G, Nawaz S, Title LM, Gau G, Blaustein AS, Booth DC, Bates ER, Spertus JA, Berman DS, Mancini GB, Weintraub WS, COURAGE Trial Research Group (2007). Optimal medical therapy with or without PCI for stable coronary disease. N Engl J Med.

[37] Gao L, Wang Y, Liu Y, Cao F, Chen Y (2017). Long-term clinical outcomes of successful revascularization with drug-eluting stents for chronic total occlusions: a systematic review and meta-analysis. Catheter Cardiovasc Interv.

[38] Ma Y, Li D, Li J, Li Y, Bai F, Qin F, Zhou S, Liu Q (2018). Percutaneous coronary intervention versus optimal medical therapy for patients with chronic total occlusion: a meta-analysis and systematic review. J Thorac Dis.

[39] Pica S., Di Giovine G., Bollati M., Testa L., Bedogni F., Camporeale A., Pontone G., Andreini D., Monti L., Gasparini G., Grancini L., Secco G.G., Maestroni A., Ambrogi F., Milani V., Lombardi M. (2018). Cardiac magnetic resonance for ischaemia and viability detection. Guiding patient selection to revascularization in coronary chronic total occlusions: The CARISMA_CTO study design. International Journal of Cardiology.

[40] Merinopoulos I, Corballis N, Eccleshall SC, Vassiliou VS (2018). Risk of sudden cardiac death: are coronary chronic total occlusions an additional risk factor?. World J Cardiol.

