# Supplementary figures and images for: Percutaneous Coronary Intervention Versus Medical Therapy for Chronic Total Occlusion of Coronary Arteries: A Systematic Review and Meta-Analysis
Source: Curr Atheroscler Rep. 2019 Aug 9;21(10):42. doi: 10.1007/s11883-019-0804-8 (PMC6689032; doi:10.1007/s11883-019-0804-8)

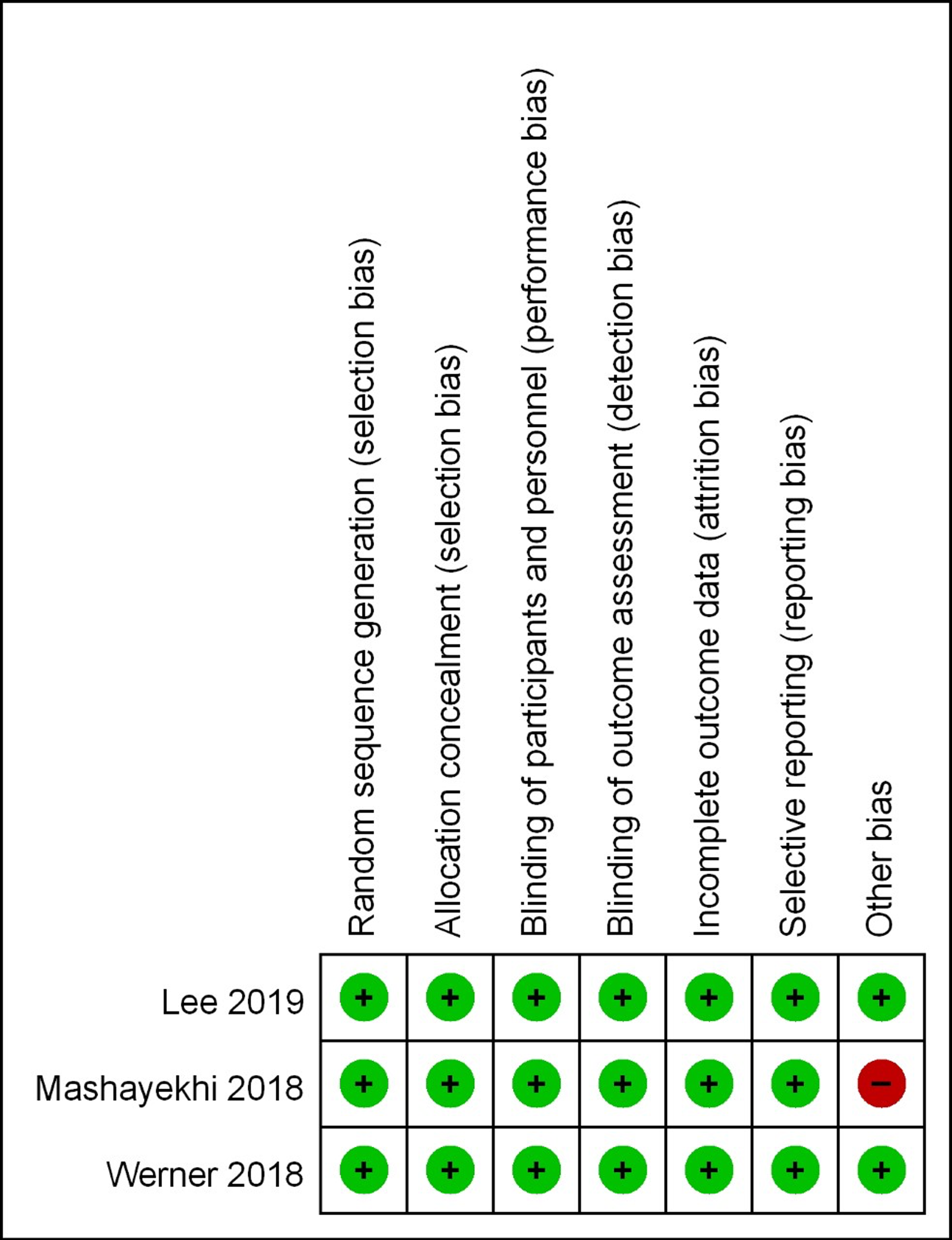

Supplement: Supplementary file 1 — Cochrane risk of bias assessment for included randomized controlled trials. (PNG 2742 kb) [file 11883_2019_804_Fig4_ESM.png]

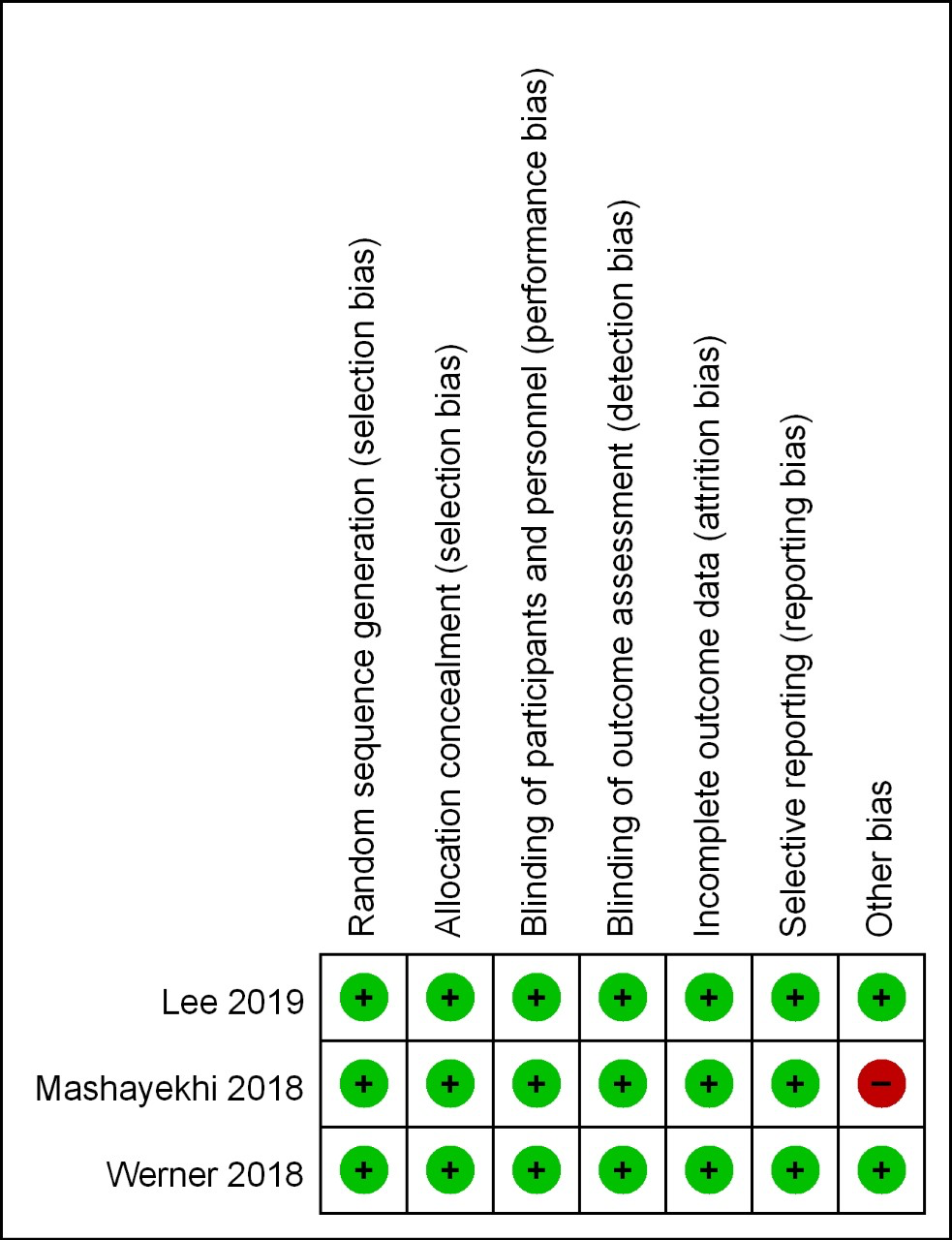

Supplement: Supplementary file 2 — High resolution image (TIFF 4918 kb) [file 11883_2019_804_MOESM1_ESM.tiff]
