# Supplementary material for: Percutaneous Coronary Intervention Versus Medical Therapy for Chronic Total Occlusion of Coronary Arteries: A Systematic Review and Meta-Analysis
Source: Curr Atheroscler Rep. 2019 Aug 9;21(10):42. doi: 10.1007/s11883-019-0804-8 (PMC6689032; doi:10.1007/s11883-019-0804-8)
Supplement: Supplementary file 3 — (DOCX 24 kb) [file 11883_2019_804_MOESM2_ESM.docx]

Supplementary Table 1. The Newcastle–Ottawa Quality Assessment Scale risk of bias scale for included cohort studies

|  |  | Selection |  |  |  |  | Outcome |  |  |
| --- | --- | --- | --- | --- | --- | --- | --- | --- | --- |
| Studies | Representativeness of the exposed cohort | Selection of the non-exposed cohort | Ascertainment of exposure | Outcome of interest not present at start of study | Comparability | Assessment of outcome | Adequacy of duration of follow-up | Adequacy of completeness of follow-up | Total score  (0-9) |
| Song 2011 | 1 | 1 | 1 | 0 | 0 | 1 | 1 | 0 | 5 |
| Fujino 2014 | 1 | 1 | 1 | 0 | 0 | 1 | 1 | 0 | 5 |
| Kim 2015 | 1 | 1 | 1 | 0 | 0 | 1 | 1 | 1 | 6 |
| Jang 2015 | 1 | 1 | 1 | 0 | 0 | 1 | 1 | 1 | 6 |
| Shuvy 2017 | 1 | 1 | 1 | 1 | 1 (age) | 1 | 1 | 1 | 8 |
| Tomasello 2015 | 1 | 1 | 1 | 1 | 0 | 1 | 1 | 1 | 7 |
| Ladwiniec 2015 | 1 | 1 | 1 | 1 | 0 | 1 | 1 | 1 | 7 |
| Yang 2016 | 1 | 1 | 1 | 1 | 0 | 1 | 1 | 1 | 7 |
| Choi 2017 | 1 | 1 | 1 | 1 | 0 | 1 | 1 | 1 | 7 |
| Guo 2018 | 1 | 1 | 1 | 1 | 1 (age) | 1 | 1 | 1 | 8 |
| Yuste 2017 | 1 | 1 | 1 | 1 | 0 | 1 | 1 | 0 | 6 |
| Ungvari 2011 | 1 | 1 | 1 | 0 | 0 | 1 | 1 | 0 | 5 |
| Rha 2018 | 1 | 0 | 1 | 0 | 0 | 1 | 1 | 0 | 4 |
| Choo 2019 | 1 | 1 | 1 | 1 | 0 | 1 | 1 | 1 | 7 |
